# SUMOylation of Paraflagellar Rod Protein, PFR1, and Its Stage-Specific Localization in *Trypanosoma cruzi*

**DOI:** 10.1371/journal.pone.0037183

**Published:** 2012-05-17

**Authors:** Takeshi Annoura, Takashi Makiuchi, Idalia Sariego, Takashi Aoki, Takeshi Nara

**Affiliations:** Department of Molecular and Cellular Parasitology, Juntendo University School of Medicine, Bunkyo-ku, Tokyo, Japan; University of Texas-Houston Medical School, United States of America

## Abstract

**Background:**

The flagellate protozoan parasite, *Trypanosoma cruzi*, is a causative agent of Chagas disease that is transmitted by reduviid bugs to humans. The parasite exists in multiple morphological forms in both vector and host, and cell differentiation in *T. cruzi* is tightly associated with stage-specific protein synthesis and degradation. However, the specific molecular mechanisms responsible for this coordinated cell differentiation are unclear.

**Methodology/Principal Findings:**

The SUMO conjugation system plays an important role in specific protein expression. In *T. cruzi*, a subset of SUMOlylated protein candidates and the nuclear localization of SUMO have been shown. Here, we examined the biological roles of SUMO in *T. cruzi*. Site-directed mutagenesis analysis of SUMO consensus motifs within *T. cruzi* SUMO using a bacterial SUMOylation system revealed that *T. cruzi* SUMO can polymerize. Indirect fluorescence analysis using *T. cruzi* SUMO-specific antibody showed the extra-nuclear localization of SUMO on the flagellum of epimastigote and metacyclic and bloodstream trypomastigote stages. In the short-flagellate intracellular amastigote, an extra-nuclear distribution of SUMO is associated with basement of the flagellum and becomes distributed along the flagellum as amastigote transforms into trypomastigote. We examined the flagellar target protein of SUMO and show that a paraflagellar rod protein, PFR1, is SUMOylated.

**Conclusions:**

These findings indicate that SUMOylation is associated with flagellar homeostasis throughout the parasite life cycle, which may play an important role in differentiation of *T. cruzi*.

## Introduction

Chagas disease, caused by infection with the parasitic protist, *Trypanosoma cruzi*, is one of important neglected tropical diseases endemic in Central and South America that burdens 16–18 million people with 25,000 of annual deaths. *T. cruzi* includes multiple developmental forms in its life cycle. In the insect vector, triatomine bug, epimastigote proliferates and transforms into metacyclic trypomastigote, which is transmitted via triatomine urine and invades inside the human cell [1]. After invasion, metacyclic trypomastigote transforms into the round-shaped amastigote, which shorten the flagellum and multiplies continuously until the host cell dies. Prior to the rupture of died cell, amastigote extends the flagellum and transforms back into infective bloodstream trypomastigote.

This reciprocal transformation cycle is particularly important for pathogenesis of Chagas disease because trypomastigote cannot propagate. Although transformation is accompanied with a variety of morphological and metabolic changes [2], [3], the molecular mechanisms required for such differentiation and proliferation remain to be uncovered [4].

Post-translational modifications play an important role in the functional expression of proteins by altering their stability, activity, and localization, as well as their ability to interact with other molecules. SUMO, the small ubiquitin-related modifier, is known to play an important role in a wide variety of eukaryotic cellular processes by modifying numerous proteins and modulating their function and/or activation [5]. SUMO conjugation is essential in eukaryotes and regulates specific protein expression, often by antagonizing ubiquitin-mediated protein degradation, and the downstream effects include cell cycle progression, DNA repair and stress responses [6], [7].

The presence of the SUMO conjugation system in trypanosomatid parasites has been recently reported. In *T. brucei*, SUMO is essential in cell cycle regulation in procyclic and bloodstream forms [8]. On the other hand, topoisomerase-II, which accumulates at centromere during prometaphase and is required for regulated chromosome segregation, is not modified by SUMO in *T. brucei*, despite of its regulation via SUMO conjugation in other eukaryotes [9]–[11]. Bayona *et al.* have recently reported the occurrence of SUMO conjugation in *T. cruzi* [12]. Proteomic analysis of the SUMOylated protein-enriched fraction showed that 236 candidate proteins appeared to be SUMOylated. Among them, metacaspase 3 was found to be a *bona fide* substrate of SUMOylation. However, the biological roles of SUMO conjugation in trypanosomatids are still unclear.

Several SUMOs (SMT3 and human SUMO2 and SUMO3) can polymerize and become what is known as poly-SUMO via the N-terminal region of the SUMO consensus motif (ψ-K-x-D/E, where ψ is a hydrophobic residue, K is the lysine conjugated to SUMO, x is any amino acid, and D and E are acidic residues). The biological importance of poly-SUMOylation is strongly implicated in yeast strains lacking poly-SUMO activity [13].

In the present study, we report the occurrence of polymerization of *T. cruzi* SUMO and its involvement in the flagellar homeostasis of the parasite. We show the presence of SUMO consensus motifs in *T. cruzi* SUMO and the occurrence of poly-SUMOylation using a *Escherichia coli*-based *in vivo* chimeric SUMOylation system. Indirect immunofluorescence analysis (IFA) demonstrates the localization of SUMO in the nucleus in all parasite developmental stages. In addition, an extra-nuclear localization of SUMO is associated with the parasite flagellum; in the intracellular amastigote, SUMO is associated with basement of the flagellum and becomes clearly distributed along the flagellum as amastigote transforms into trypomastigote. Analysis using the bacterial SUMOylation and Western blots of the parasite extracts revealed that a paraflagellar rod protein, PFR1 is one of SUMOylation substrates. The physiological roles of *T. cruzi* SUMO conjugation in flagellar homeostasis are discussed.

## Results

### The Occurrence of Poly-Sumoylation In *T. Cruzi* SUMO

**Figure 1:**
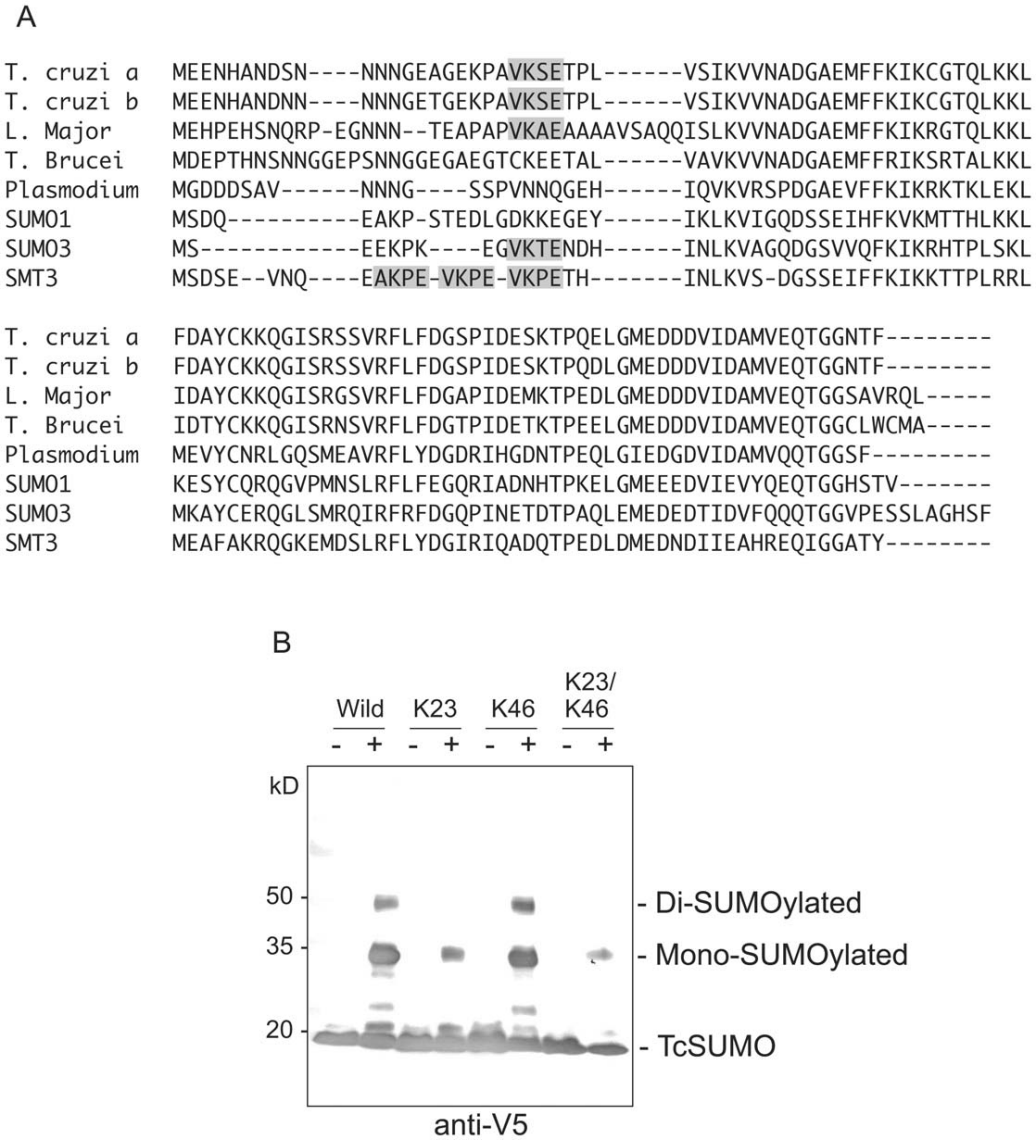
Characterization of *T. cruzi* SUMO. (**A**) Multiple alignment of the amino acid sequences of SUMOs of parasitic protists, *T. cruzi*, *L. major*, *T. brucei* and *Plasmodium falciparum*, SUMO1 and SUMO3 of human, and SMT3 of *Saccharomyces cerevisiae*. Gaps are shown as dashes. Shaded amino acids indicate the putative poly-SUMO motifs. (**B**) Western blot analysis of poly-SUMOylation of *T. cruzi* SUMO using an *E. coli* SUMOylation system. The recombinant TcSUMO tagged with His-V5 at the N-terminal (Wild) and a series of site-directed mutants were expressed in *E. coli* in the absence (−) or presence (+) of human SUMO1 and its modifiers and reacted with anti-V5 antibody. K23, K46, and K23/K46 indicate the mutants lacking the respective Lys residues.

Two SUMO genes, *TcSUMOa* and *TcSUMOb*, are present in the *T. cruzi* genome sequence database (designated *TcSUMOa* and *TcSUMOb*; Tc00.1047053511661.50 and Tc00.1047053507809.70, respectively) [14]. The amino acid sequence alignment and the putative poly-SUMO motifs of various SUMOs are shown in Fig. 1A. The strength of the predicted poly-SUMO motif was calculated using SUMOplot™ Analysis Program (http://www.abgent.com.cn/doc/sumoplot/login.asp). The predicted probability scores for *T. cruzi* SUMOs are 0.99 and 0.77 for ^22^VKSE and ^45^IKCG, respectively. Notably, the presence of poly-SUMO motif varies among protozoan parasites. The poly-SUMO motif is present in *T. cruzi* and *L. major* SUMOs but absent in *T. brucei*, despite of significant conservation of the amino acid sequence in trypanosomatid SUMOs.

To determine whether the poly-SUMO motifs of *T. cruzi* SUMO is SUMOylated, we used an *Escherichia coli-*based, *in vivo* chimeric SUMOylation system [15], [16]. This system is able to detect SUMOylation of heterologous eukaryotic substrate proteins by conjugation of the human SUMOylation machinery [17]. We expressed the recombinant TcSUMO tagged with His-V5 at its N-terminal in *E. coli* together with human SUMO1 (hSUMO1) and its modifying enzymes, E1 and E2. In this system, TcSUMO only serves as a substrate because the expressed TcSUMO is an inactivated form. We expressed TcSUMO and a series of the site-directed mutants, of which the putative SUMOylation site, Lys23 or Lys46, was replaced with Arg independently or in combination (Fig. 1B, K23R, K46R, or K23R/K46R mutant).

Western blot analysis using anti-V5 antibody showed the multiple bands of 20-, 35-, and 50-kDa in wild type TcSUMO and K46R mutant (Fig. 1B). Because the recombinant TcSUMO and hSUMO1 are 20-kDa and 15-kDa proteins, respectively, the 35- and 50-kDa bands corresponded to mono- and di-SUMO1-conjugated TcSUMO, respectively. In contrast, the K23R and K23R/K46R mutants showed loss of the 50-kDa band, indicating loss of SUMOylation by K23R mutation. The 50-kDa band appeared to correspond to “di”-SUMO1-conjugated TcSUMO, suggesting the occurrence of SUMOylation at least two sites; however, an additional SUMOylation site was unclear. We conclude that Lys23 is involved in poly-SUMOylation in this system.

### SUMOylation in the Developmental Stages of *T. Cruzi*

We examined SUMOylation in different developmental stages of *T. cruzi* (Supporting Fig. S1). Western blot analysis using the anti-TcSUMO antibody was carried out to detect SUMOylated proteins, which are covalently bound to SUMO between the ε-amino group of lysine of the substrate proteins and the C-terminal carboxyl group of SUMO. The specificity of anti-TcSUMO antibody was confirmed by Western blot using the *E. coli* lysate expressing recombinant TcSUMO, showing a single band of expected size.

Numerous bands were detected, consistent with the recent report [12], in all developmental stages; most bands were common but several bands were specific to the developmental stages. These results corroborate previous data [12] that not only SUMOylation occur in all *T. cruzi* developmental stages but also some proteins are specifically SUMOylated within each developmental stage.

Using immunoprecipitation (IP) techniques, we attempted to isolate SUMOylated parasite proteins in order to determine the physiological substrates of SUMO conjugation. IP of epimastigote cell lysates using anti-TcSUMO antibodies followed by Western blotting showed a strong band at 15 kDa, which corresponds to TcSUMO monomer; however, we could not detect any substrate proteins (data not shown).

We suspected that the failure of the detection of SUMOylated proteins in the parasite lysate might be due to the rapid turnover of SUMO conjugation. SUMO-specific cleaving cysteine proteases (SENP, sentrin proteases) is not only responsible for activation of SUMO, by which a C-terminal GG motif is cleaved off, but also mediates removal of SUMO from the SUMOylated proteins. To clarify this, we expressed the recombinant SUMO tagged with N-terminal FLAG and C-terminal MYC in *T. cruzi* epimastigotes and examined the SENP activity of the parasite. We failed to detect the unprocessed forms of SUMO after immunoprecipitation. Cleavage (activation) of the immature SUMO were not inhibited even in the presence of N-ethyl-maleimide (NEM), a canonical SENP inhibitor that is an alkylating agent and modifies all cysteine residues, at high concentrations (Supporting Fig. S2A).

Overexpression of the recombinant SUMO (FLAG-TcSUMO-MYC) appeared to be toxic to the parasite. This transgenic parasite did not show the defect of growth, but showed morphological abnormality, probably due to the physiological disturbance caused by the excess amount of SUMO (Supporting Fig. S2B).

### Localization of SUMO in *T. Cruzi* Nucleus and Flagellum

**Figure 2:**
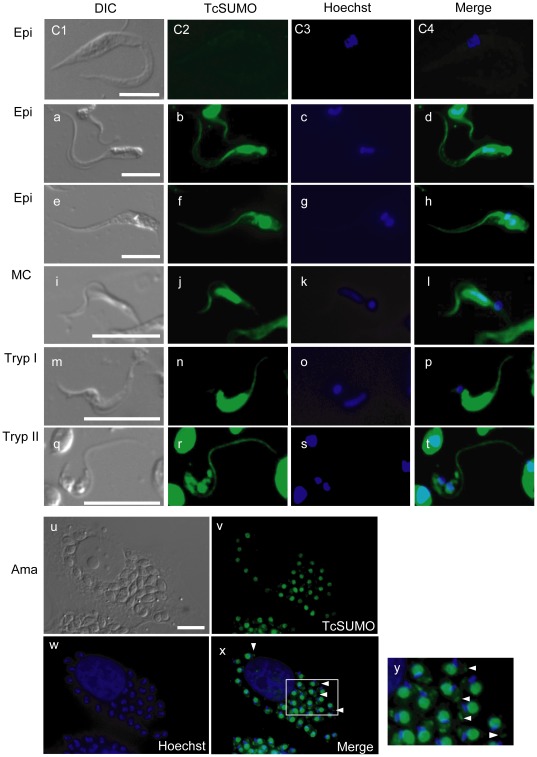
Localization of SUMO in the life cycle of *T. cruzi*. Indirect immunofluorescence analysis using anti-TcSUMO antibody of epimastigotes (Epi; a–h), metacyclic trypomastigotes (MC; i–l), trypomastigotes of Type I (Tryp I; m–p, see text) and Type II (Tryp II; q–t), and amastigotes (Ama; u–y). The parasite nucleus and kinetoplast were counterstained with Hoechst 33342. As a negative control, epimastigotes were reacted with the labeled pre-immune serum (C1–C4). Labels are as follows; DIC, differential interference contrast images (left column); TcSUMO, parasites labeled with anti-TcSUMO antibody (next left); Hoechst, parasites stained with Hoechst 33342 (center right); and Merge, merged images (right). Panel y shows the magnified image of the inset in panel x. Scale bar = 10 µm.

The subcellular localization of SUMO in *T. cruzi* was examined by IFA using anti-TcSUMO antibody (Fig. 2). Pre-immune sera, as well as the antibody fraction depleted of anti-TcSUMO, did not react with the parasites, confirming the specificity of anti-TcSUMO antibody (Fig. 2, C1–C4, top row). Strong fluorescence signal was observed predominantly in the nucleus in all parasite developmental stages, consistent with the recent findings [12].

Unexpectedly, the signal was also detected in the flagellum of both epimastigotes (Fig. 2a–h) and metacyclic trypomastigotes (Fig. 2i–l). The intensity and pattern of antibody staining in the nucleus of trypomastigotes was of two types: Type I is characterized by strong intensity of anti-SUMO staining along an elongated nucleus (Fig. 2m–p); and Type II is typified by weak intensity staining around a round nucleus (Fig. 2q–t). 80% of trypomastigotes examined so far appeared to have type I staining and the reminder having type II, whereas the flagellum was clearly and similarly labeled in both types (Fig. 2t).

In intracellular amastigotes, SUMO was also detected in the nucleus, as well as small ‘dot’-like staining at the basement of the flagellum (Fig. 2u–y, shown in arrowheads). These results indicate that SUMO is localized not only in the nucleus but also the flagellum and strongly suggests that SUMOylation takes place in both these organelles in *T. cruzi*.

### A Paraflagellar Rod Protein, PFR1, is SUMOylated in *T. Cruzi*

To identify the *T. cruzi* flagellum protein target(s) of SUMOylation, we screened *in silico* for flagellum-associated proteins that are expressed in all developmental stages and possess SUMO consensus motif. Consequently, we identified a paraflagellar rod protein, PFR1 by *in silico* screening, which contains three putative SUMO consensus motifs (^54^LKAE, ^136^AKME, and ^477^MKKE; see Supporting Fig. S3). Interestingly, proteomic analyses revealed the expression of PFR1 also in the “short-flagellate” amastigotes [18]. In addition, PFR proteins are found to be degraded by proteasome in *T. cruzi* [19] and it is important to note that SUMOylated proteins can serve as substrates for ubiquitin-proteasome degradation.

**Figure 3:**
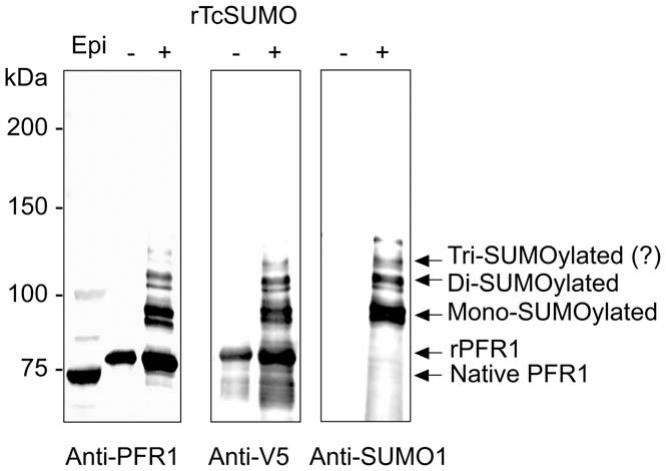
SUMOylation of a flagellar rod protein, PFR1, in *T. cruzi*. Western blot analysis of *T. cruzi* PFR1 using anti-PFR1, anti-V5 and anti-SUMO1 antibodies. The cell lysates of epimastigotes (Epi, 1×10^7^ parasites/lane) and *E. coli* expressing His-V5 tagged PFR1 (rPFR1) in the absence (−) or presence (+) of human SUMO1 and its modifiers were separated by SDS-PAGE and reacted with anti-PFR1, anti-V5 or anti-SUMO1 antibody. Note that the bands for SUMOylated rPFR1, which corresponded to the mono-, di- or tri-SUMOylated proteins, were only detectable in the presence of human SUMO1 and its modifiers.

To examine whether PFR1 is SUMOylated in *T. cruzi*, we generated an antibody raised against a synthetic TcPFR1 peptide. Western blot analysis of epimastigote cell lysate using anti-PFR1 antibody showed clearly three bands, a strong 70-kDa and weak 85- and 100-kDa bands, which are likely to correspond to the intact PFR1 and mono- and di-SUMOylated PFR1, respectively (Fig. 3, lane 1).

We further examined the SUMOylation of PFR1 using an *E. coli*-based *in vivo* chimeric SUMOylation system. Recombinant PFR1 tagged with His-V5 was expressed in *E. coli*, either in the presence or absence of SUMO1 and its modifiers, and purified using a His-affinity column. In the absence of SUMO1, the recombinant PFR1 was detected as a single band of 75-kDa using both anti-V5 and PFR1 antibodies (Fig. 3, lanes 2 and 4; −lanes respectively). On the other hand, both anti-V5 and PFR1 antibodies reacted with the additional bands at 90 kDa, 105 kDa, and 120 kDa in the presence of SUMO1, which corresponded to mono-, di-, and tri-SUMOylated PFR1, respectively, (Fig. 3, lanes 3 and 5; +lanes respectively). Consistently, Western blot using anti-SUMO1 antibody showed the same patterns in the presence of SUMO1 (Fig. 3, lane 7). These results suggest that PFR1 is a substrate of SUMO and is likely multi-SUMOylated.

To know the SUMOylation site(s) of TcPFR1, we constructed and analyzed a series of the V5-tagged, site-directed TcPFR1 mutants, of which Lys55, Lys137 and Lys478 in the putative SUMO consensus motifs were replaced with Arg individually or in combination. Western blot analysis was carried out repeatedly using both anti-V5 and SUMO1 antibodies; however, we could not obtain the clear results (data not shown). Therefore, the exact Lys residue(s) of PFR1 for SUMOylation was unclear using this expression system.

### Synchronization of SUMOylation of PFR1 and Flagellar Elongation in *T. Cruzi* Amastigote

**Figure 4:**
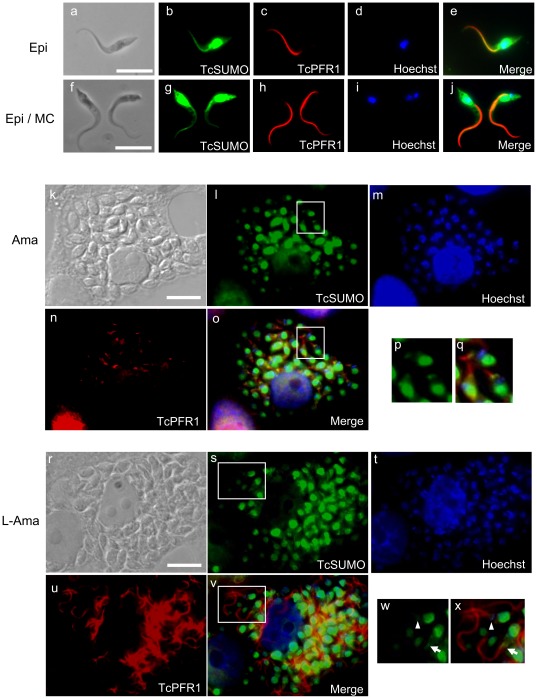
Co-localization of SUMO and PFR1 during developmental stages of *T. cruzi*. Immunofluorescence analysis of epimastigotes (Epi, a–e, and a parasite on the left in f–j), metacyclic trypomastigotes (MC; a parasite on the right in f–j), amastigotes (Ama; k–q), and amastigotes transforming to trypomastigotes (T-Ama; r–x), using anti-*T. cruzi* SUMO (TcSUMO, green) and anti-PFR1 (TcPFR1, red) antibodies. The nucleus and kinetoplast were counterstained with Hoechst 33342 (blue). DIC, differential interference contrast images; Merge, merged images. Panels p, q, w, and x correspond to the magnified image of the inset in panel i, o, s, and v, respectively. Scale bar = 10 µm.

SUMO and PFR1 were found to co-localize along with flagellum, along its whole length, in both epimastigotes and metacyclic trypomastigotes (Fig. 4a–j). However, localization of SUMO did not merge fully with that of PFR1 in amastigotes (Fig. 4k–q). The distribution of SUMO is limited to the base of the flagellum, presumably the flagellar pocket, whereas PFR1 appears to localize along the length of the very short flagellum (Fig. 4p–q), suggesting a dissociation of SUMO from PFR1 in amastigotes.

We further examined whether the localization of SUMO correlates with intracellular development of amastigote as it propagates and then differentiates into trypomastigote inside the host cell. We therefore focused on the intracellular transition of amastigote to trypomastigote, specifically when the flagellum becomes elongated characteristic of the trypomastigote stage while the parasite body retains its amastigote oval shape (Fig. 4r–x). We found that SUMO was localized from the base of flagellum and extended along with the elongated flagellum (Fig. 4w–x). These results indicate that SUMOylation of PFR1 correlates with elongation of flagellum during transformation from amastigote to trypomastigote, suggesting the importance of SUMO conjugation in differentiation of the parasite.

## Discussion

In the present study, we show the occurrence of poly-SUMOylation of SUMO and the stage-specific localization of SUMO on the parasite nucleus and flagellum in *T. cruzi*. Furthermore, we demonstrate that PFR1 is a flagellar substrate of SUMO, indicating the regulation of flagellar homeostasis by the SUMO conjugation system in *T. cruzi*. This is the first report demonstrating the occurrence of SUMOylation for the flagellar components.

Some SUMO proteins, including human SUMO2 and SUMO3, and yeast SMT3, can polymerize via a N-terminal SUMOylation consensus motif within SUMO itself (see Fig. 1) [13]. Nevertheless, the biological significance of poly-SUMO is still unclear. Amino acid sequence comparisons of various protozoan parasite SUMOs revealed the presence of poly-SUMO motif in *T. cruzi* and *L. major* but not in *T. brucei* and *P. falciparum*. These suggest that *T. brucei* and *P. falciparum* SUMOs are likely to be involved in the simple SUMOylation system, different from that of *T. cruzi* or *L. major* having the poly-SUMO motif.

We examined using a bacterial expression system whether the putative SUMO consensus motif of *T. cruzi* SUMO is SUMOylated. Site-directed mutagenesis of Lys23 in the poly-SUMO motif (^22^VKSE) resulted in loss of the SUMOylated product, suggesting that this motif participates in poly-SUMOylation. On the other hand, another putative poly-SUMO motif (^45^IKCG) was not SUMOylated in this system, whereas the unexpected SUMOylation was detected.

SUMOylation affects many fundamental pathways in both nucleus and cytoplasm [20]. These processes encompass a variety of basic cellular and metabolic functions, including ion transport, mitochondrial fission and fusion, and protein transport along the axons of neurons [7], [21], [22]. In the present study, we demonstrated localization of SUMO not only in the nucleus but also on the flagellum of *T. cruzi*. In trypanosomatids, regulation of flagellar homeostasis is a key biological activity and correlates tightly with their cell cycle [23]. During cell division in trypanosomatids, synthesis of a daughter flagellum is preceded by duplication of cellular components such as basal body and mitochondrion, followed by segregation of cells. Importantly, SUMOylation is known to play critical roles in the G2/M phase in yeast [5]. In trypanosomatids, this phase is accompanied by synthesis of the flagellum [23]. Therefore, it is highly likely that SUMO is involved in the maintenance of flagellar homeostasis in coordination with the timing of cell division in *T. cruzi*.

By *in silico* search for the flagellar proteins carrying a SUMOylation motif, we found that PFR1, a component of the paraflagellar rod, possesses putative SUMOylation motifs. Recent proteomic analysis has also suggested PFR1 to be a potential SUMOylated protein [12]. Western blot analysis of *T. cruzi* epimastigotes using anti-PFR1 antibody showed three bands for PFR1, which correspond to the bands of the non-conjugated, mono- and di-SUMOylated PFR1 in size. In addition, Western blot analysis of the V5-tagged recombinant PFR1 using anti-PFR1 and anti-V5 antibodies showed the thoroughly identical patterns, suggesting that PFR1 is SUMOylated.

Identification of Lys residue(s) responsible for SUMOylation of PFR1 was difficult. Site-directed mutagenesis of the putative SUMOylation motifs (^55^LKAE, ^137^AKME, and ^478^MKKE, see Fig. S2) did not show the clear results. Bayona *et al.* [12] reported that approximately 60% of the potential SUMOylated proteins screened by proteome analysis did not have canonical SUMOylation motifs [12]. In agreement with this report, our failure of identifying SUMOylation sites of PFR1 might be attributable to the presence of the non-canonical SUMOylation motifs.

As for the physiological role of SUMO conjugation, we demonstrated the localization of SUMO and PFR1 in both extracellular and intracellular parasites. In the intracellular short-flagellate amastigotes, the localization of SUMO is limited to a region close to the basement of the flagellum. This localization is extended along with flagellum in the late stage amastigotes and is characterized by elongated flagellum with typical oval shape. It is important to note that SUMO often acts as an antagonist of ubiquitin. It has been reported that TcPFRs are degraded via the ubiquitin–proteasome pathway [19]. Therefore, SUMOylation of flagellar proteins may counteract to the ubiquitin-dependent proteolysis in *T. cruzi*, preventing the degradation of flagellar components. This is in agreement with the finding that SUMO is co-localized with PFR1 once parasites initiate flagellum formation.

SUMO can also act in concert with ubiquitin. Several proteins, which are poly-SUMOylated with SUMO2 or SUMO3, are found to be ubiquitinated and subsequently degraded by proteasome [24], [25]. In order to clarify the physiological relationship between SUMO and ubiquitin in flagellar homeostasis of the parasite, it is important to determine whether SUMOylation of PFR1 is the consequence of SUMOylation at multiple sites or polymerization of SUMO at one site.

Failure of demonstration of SUMOylated proteins in the present study is probably due to decoupling of SUMO from its substrates by SENP during experimental procedures such as immunoprecipitation. The inability of NEM to inhibit de-coupling of SUMO is likely due to physical limitations of the experimental procedures. Recently, Ponder *et al.* reported small molecule inhibitors for *P. falciparum* SENP, which inhibited not only recombinant PfSENP1 activity but also replication of erythrocytic stage of *P. falciparum* [26].

In conclusion, our findings provide new insights into a *T. cruzi* SUMO conjugation system and also identify potential physiological roles for TcSUMO in nuclear and flagellar homeostasis throughout the all the parasite life cycle. Clearly, an understanding of SUMO and the variety of SUMO regulated processes will shed light on the molecular mechanisms controlling parasite survival, cell proliferation and differentiation.

## Materials and Methods

### Parasites and Host Cells

The epimastigote of *T. cruzi* Tulahuen strain was maintained in LIT medium as described previously [27]. HeLa cells, a human cervical carcinoma cell line [28], were generously gifted to TAo by Dr. Yoshio Kaneda of Tokai University [29]. HeLa cells were used and maintained as a host of the mammalian stages of *T. cruzi* as described [30], [31]. Briefly, HeLa cells (1–3×10^5^ cells) were infected by *T. cruzi* trypomastigotes (1–5×10^5^ parasites) in MEM (Sigma-Aldrich Japan, K.K., Japan) supplemented with 10% fetal bovine serum (FBS) in 25-cm^2^ culture flasks and subcultured every 2–3 days. Tissue-cultured trypomastigotes and amastigotes were purified as described with minor modifications [31]. Briefly, extracellular parasites were collected in 50-ml polypropylene tubes and settled at 37°C for 4 h. The motile trypomastigotes were purified from the top of the medium, and the sedimented parasites were confirmed to be extracellular amastigotes by microscopic observation.

### Cloning of *T. Cruzi* SUMO and PFR1 Genes

Genes for *T. cruzi SUMO* (TcSUMO) and *PFR1* (TcPFR1) (accession numbers: Tc00.1047053511661.50+Tc00.1047053507809.70 and Tc00.1047053509617.20 respectively) were cloned by PCR using KOD-plus-DNA polymerase (High-Fidelity type, Toyobo Co. Ltd., Japan), the genomic DNA of *T. cruzi* Tulahuen as a template, and the specific primers (Sense, 5′-CACCATGGAGGAGAATCATGCAAATGAC-3′, and antisense, 5′-TCACCCGCCTGTCTGCTCAACCA-3′, for TcSUMO; sense, 5′-CACCATGTCTGCCGAGGAAGCCAC-3′, and antisense, 5′-TCACTCGAGGCGGGCGGGGGC-3′, for TcPFR1). The PCR product was cloned in a directional entry vector, pENTR/D-TOPO, or a directional expression vector, pET151/D-TOPO, (Invitrogen), and sequenced using an automated DNA sequencer.

### Antigens and Antibodies

The recombinant plasmids, pET151/TcSUMO and pET151/TcPFR1, carrying *TcSUMO* and *TcPFR1* genes, respectively, were used to transform BL21 Gold (DE3) competent cells (Stratagene). The corresponding recombinant proteins were expressed and purified under the conditions recommended by the manufacturer. The purity of each recombinant protein was confirmed by SDS-PAGE to be greater than 95%. Female Japanese white rabbits and female BALB/c mice were immunized subcutaneously with the recombinant TcSUMO or the synthetic TcPFR1 and with the recombinant TcPFR1 as described previously [32]. Polyclonal antisera were applied to HiTrap Protein G HP (for mouse serum, GE Healthcare) or HiTrap Protein A HP (for rabbit serum, GE Healthcare) column for purification of the antigen-specific IgG fractions. For production of the anti-TcPFR1 peptide antibody, a synthetic peptide, N-CLRTGGGGSGEQPRIGNNTA-C, was cross-linked with keyhole limpet hemocyanin at N-terminal cysteine, and used for immunization (BioGate Co., Ltd., Gifu, Japan). IgG specific to the TcPFR1 peptide was purified using epoxy-activated Sepharose 6B affinity chromatography (GE Healthcare) coupled with the peptide, and used for further experiments.

### Western Blotting

*T. cruzi* of each developmental stage were collected by centrifugation at 3000 rpm for 10 min at 4°C and washed with ice-cold phosphate-buffered saline (PBS, pH7.2). The purified parasites were lysed in Laemmli's sample buffer at 95°C for 10 min, followed by brief sonication. Proteins were separated by SDS-PAGE and blotted onto PVDF membranes, blocked in 2% FCS plus 0.05% Tween-PBS (PBST). The membrane was incubated with the specific antibody diluted to 1∶1000–2000 in 2% FCS plus PBST overnight at 4°C and subsequently with alkaline phosphatase-conjugated anti-rabbit or anti-mouse IgG antibody (1∶3000 each; Bio-Rad Laboratories). Chemi-luminescent detection of specific hybridization was performed with CSPD (Roche).

### Indirect Immunofluorescence Analysis

Immunofluorescence analysis of flagellar proteins was performed as described previously [33], [34]. Live epimastigotes, metacyclic trypomastigotes, and trypomastigotes were immobilized on poly-L-lysine-coated slides. HeLa cells infected with *T. cruzi* amastigotes were grown on glass coverslips in wells of 12-well plates as described previously [31]. Immunofluorescence analysis of flagellar proteins was performed as described previously [33], [34]. The parasites and host cells were fixed with paraformaldehyde for 30 min and permeabilized with 1% NP-40 in PEME (100 mM PIPES, pH 6.9, 2 mM EGTA, 1 mM MgSO_4_, 0.1 mM EDTA) [35]. Blocking was performed in blocking solution (PEME containing 1% NP-40 and 1% BSA) for 30 min, and cells were reacted with anti-TcSUMO (rabbit polyclonal antibody, 1∶100 in blocking solution) and anti-TcPFR1 (mouse polyclonal antibody, 1∶100 in blocking solution) antibodies. After washing three times with 1% NP-40 in PEME, the cells were stained with Alexa Fluor 488-conjugated anti-rabbit IgG (Invitrogen; 1∶200 in blocking solution) and Texas Red-conjugated anti-mouse IgG (Vector Laboratories, Inc.; 1∶100 in blocking solution), washed three times with 1% NP-40 in PEME, and counterstained with Hoechst 33342 (1 µM). Finally, the cells were mounted in Permafluor (anti-fluorescent type, Beckman Coulter) and examined using a fluorescent microscope (model Axioplan 2, Carl Zeiss).

### SUMOylation Analysis in *Escherichia Coli*

SUMOylation of *T. cruzi* proteins was examined using the SUMO conjugation system in *Escherichia coli* [15], [16]. Briefly, *TcSUMO* or *TcPFR1* gene was cloned in pET151, which allows the N-terminal fusion with His-V5 sequence, and expressed in BL21 Gold (DE3) *E. coli* as the substrates of SUMO in the presence or absence of the human SUMO1 and the modifying enzymes E1 and E2. Expression of the recombinant proteins was induced by adding isopropyl-β-D-thiogalactopyranoside at the final concentration of 0.2 mM, followed by incubation for 12 h at 25°C. SUMOylation of the parasite proteins was assayed by detection of the occurrence of SUMO1-conjugated recombinant proteins. The recombinant parasite proteins tagged with His-V5, were affinity-purified using the HisLink spin protein purification system (Promega) and the purified proteins were subjected to SDS-PAGE and Western blotting. Detection of His-V5-tagged and SUMO1-modified proteins was performed using alkaline phosphatase-conjugated anti-V5-antibodies (Invitrogen) and anti-SUMO1 monoclonal antibody (Abgent), respectively.

### Site-Directed Mutagenesis of SUMOylation Motif

Site-directed mutagenesis was carried out to disrupt the putative SUMOylation motifs of TcSUMO (Lys 23 or Lys 46) and TcPFR1 (Lys 55, Lys 137 or Lys 478) using QuikChange® II Site-Directed Mutagenesis Kits (Stratagene). The primers used are as follows; 5′-ggagaagccagcggtaaggtccgaaaccc-3′ and 5′-gggtttcggaccttaccgctggcttctcc-3′ for TcSUMO K23, 5′-cgagatgttcttcaagatcaggtgcgggacgcag-3′ and 5′-ctgcgtcccgcacctgatcttgaagaacatctcg-3′ for TcSUMO K46, 5′-gcggtgcccacctgagggcggagg-3′ and 5′-cctccgccctcaggtgggcaccgc-3′ for TcPFR1 K55, 5′-gacaacgccattgcgaggatggagaaggtggag-3′ and 5′-ctccaccttctccatcctcgcaatggcgttgtc-3′ for TcPFR1 K137, 5′-aggcatgccgacatgaggaaggagctgtacaag-3′ and 5′-cttgtacagctccttcctcatgtcggcatgcct-3′ for K478. The reaction products were cloned in pET151 vector and applied for SUMOylation analysis.

### Expression of TcSUMO in *T. Cruzi*

TcSUMO tagged with FLAG and MYC at N- and C-termini, respectively, was expressed in *T. cruzi. TcSUMO* gene was PCR-amplified using KOD-plus-DNA polymerase (High-Fidelity type, Toyobo Co., Ltd., Osaka, Japan), pENTR/D/TcSUMO as a template, and the primers (Sense, 5′-CACCATGGACTACAAAGACGATGACGACAAGATGGAGGAGAATCATGCAAATGAC-3′, and antisense, 5′-TCAGAGATCCTCTTCTGAGATGAGTTTTTGTTCAAACGTGTTCCCGCCTGTCTGCTC-3′). The PCR product was cloned in pENTR/D-TOPO (pENTR/D/FLAG-TcSUMO-MYC). An expression vector, pTREX-Gateway, which is a pTREX-backbone plasmid and has the GATEWAY acceptor between *Xba*I and *Xho*I, sites, allowing recombination by clonase system (Invitrogen), was a generous gift from Dr. Muneaki Hashimoto at Department of Molecular and Cellular Parasitology, Juntendo University School of Medicine. pENTR/D/FLAG-TcSUMO-MYC plasmid was reacted with pTREX-Gateway under conditions recommended by the manufacturer. The transformation of *T. cruzi* epimastigotes were transformed using the derived plasmid construct, pTREX/FLAG-TcSUMO-MYC, by electroporation and cultured in the presence of G-418 as described previously [27]. The resulting transformants expressing FLAG-TcSUMO-MYC was used for the further experiments. FLAG-TcSUMO-MYC expressing parasites were lysed in 100 ml of canonical SUMOylation Immuno-precipitation (IP) buffer (RIPA; 100 mM sodium phosphate pH 7.2, 150 mM NaCl, 1% sodium dexycholate, 1% Triton X-100, and 0.1% SDS) or flagellar protein IP buffer (PEME; as describe in material and method section for indirect immunofluorescence analysis part), which containing protease inhibitor cocktail (1 mg of aprotinin and leupeptin/ml and 10 mM phenylmethylsulfonyl fluoride), 1 mM NaF, 0.4 mM sodium orthovanadate, and 3 mM NEM. The lysate was immunoprecipitated with anti-FLAG or MYC antibodies, and collected the IP products by using Protein G Magnetic Beads under the conditions recommended by the manufacturer (Protein G Magnetic Beads, New England BioLabs).

### Ethics

All animal experiments of this study were carried out in accordance with the Fundamental Guidelines for Proper Conduct of Animal Experiment and Related Activities in Academic Research Institutions under the jurisdiction of the Ministry of Education, Culture, Sports, Science and Technology (Notice No. 71, 2006), and approved by the Committee for Animal Experimentation of Juntendo University with the Approval No. 200106. The present study did not include research involving human participants, as well as non-human primates and unpublished de novo cell lines.

## Supporting Information

Figure S1 **Detection of SUMOylation proteins in each developmental stage of** ***T. cruzi*** **.** Cell lysates of epimastigotes (Epi; 2×10^6^ parasites/lane), trypomastigotes (Tryp; 5×10^6^ parasites/lane), and amastigotes (Ama; 1×10^7^ parasites/lane) were separated by SDS-PAGE and stained with Coomassie Brilliant Blue (A) or reacted with anti-TcSUMO antibody (B). The protein amount of each lane was adjusted to an approximately similar intensity of free TcSUMO (15-kDa, shown by an asterisk). Arrows indicate the stage-specific bands. As a control (C), the *E. coli* lysate expressing recombinant TcSUMO was reacted with anti-TcSUMO antibody and displayed a single band of the expected size. See also Materials and Methods.
(TIF)

Figure S2 **Activation of SUMO in** ***T. cruzi*** **epimastigote.** The recombinant SUMO tagged with the N-terminal FLAG and the C-terminal MYC (F-TcSUMO-M) was expressed in *T. cruzi*. The *T. cruzi* lysates were extracted in the presence of SENP inhibitor, NEM, and immunoprecipitated using anti-FLAG or anti-MYC antibody under the conditions for the canonical SUMOylation detection (A) or for the detection of the flagellar proteins (B). The recombinant F-TcSUMO-M (control) and the precipitated proteins were probed with either antibody. The band corresponding to F-TcSUMO (processed form) was only detected in the immunoprecipitated fractions. Note that *T. cruzi* epimastigote overexpressing F-TcSUMO-M showed morphological abnormality, in which the shape of the parasite became stumpy (C). Scale bar = 10 µm. See also Materials and Methods.
(TIF)

Figure S3 **The amino acid sequence of TcPFR1 and the potential SUMO conjugation motifs.** Gray shading indicates the putative SUMO conjugation motif with high probability. SUMOylation probability scores were calculated using the SUMOplot™ analysis program, and those with high probability are shown below the amino acid sequence. The underline indicates the C-terminal-specific sequence of PFR1, used as an antigen for a specific antibody.
(TIF)
