# Supplementary figures and images for: SUMOylation of Paraflagellar Rod Protein, PFR1, and Its Stage-Specific Localization in Trypanosoma cruzi
Source: PLoS One. 2012 May 17;7(5):e37183. doi: 10.1371/journal.pone.0037183 (PMC3355114; doi:10.1371/journal.pone.0037183)

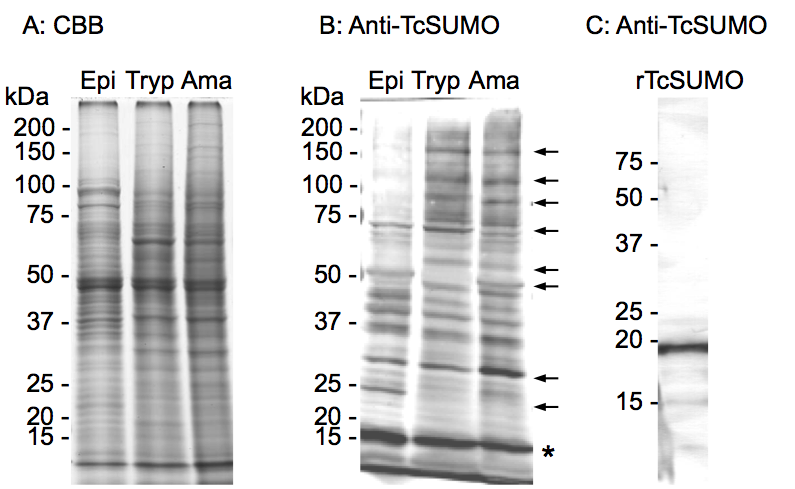

Supplement: Figure S1 — Detection of SUMOylation proteins in each developmental stage of T. cruzi . Cell lysates of epimastigotes (Epi; 2×10^6^ parasites/lane), trypomastigotes (Tryp; 5×10^6^ parasites/lane), and amastigotes (Ama; 1×10^7^ parasites/lane) were separated by SDS-PAGE and stained with Coomassie Brilliant Blue (A) or reacted with anti-TcSUMO antibody (B). The protein amount of each lane was adjusted to an approximately similar intensity of free TcSUMO (15-kDa, shown by an asterisk). Arrows indicate the stage-specific bands. As a control (C), the E. coli lysate expressing recombinant TcSUMO was reacted with anti-TcSUMO antibody and displayed a single band of the expected size. See also Materials and Methods. (TIF) [file pone.0037183.s001.tif]

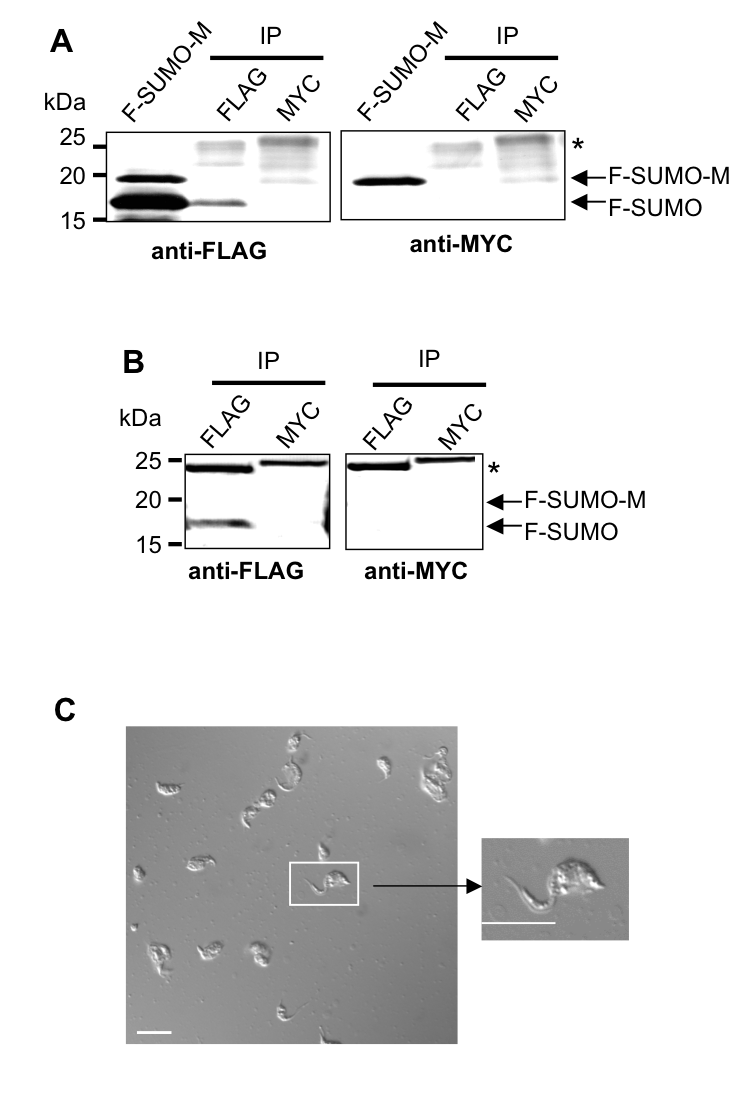

Supplement: Figure S2 — Activation of SUMO in T. cruzi epimastigote. The recombinant SUMO tagged with the N-terminal FLAG and the C-terminal MYC (F-TcSUMO-M) was expressed in T. cruzi. The T. cruzi lysates were extracted in the presence of SENP inhibitor, NEM, and immunoprecipitated using anti-FLAG or anti-MYC antibody under the conditions for the canonical SUMOylation detection (A) or for the detection of the flagellar proteins (B). The recombinant F-TcSUMO-M (control) and the precipitated proteins were probed with either antibody. The band corresponding to F-TcSUMO (processed form) was only detected in the immunoprecipitated fractions. Note that T. cruzi epimastigote overexpressing F-TcSUMO-M showed morphological abnormality, in which the shape of the parasite became stumpy (C). Scale bar = 10 µm. See also Materials and Methods. (TIF) [file pone.0037183.s002.tif]

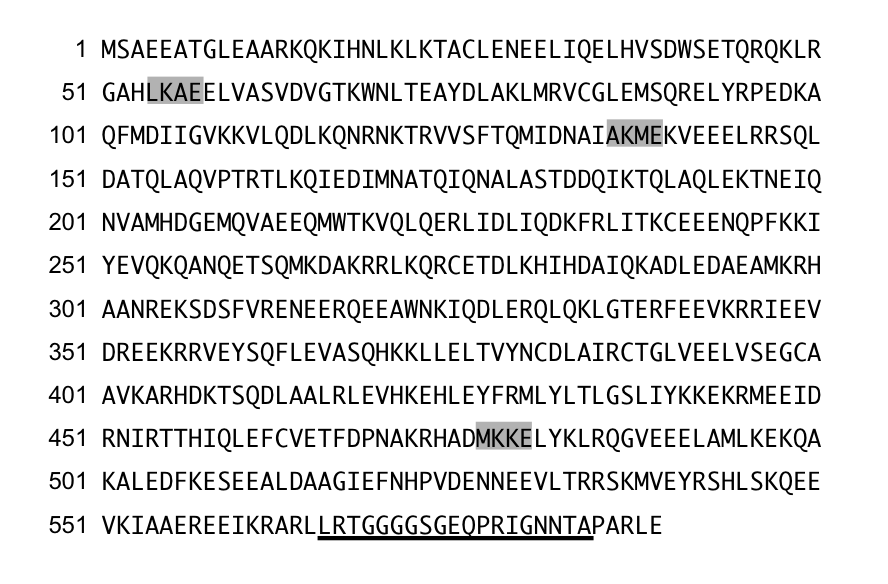

Supplement: Figure S3 — The amino acid sequence of TcPFR1 and the potential SUMO conjugation motifs. Gray shading indicates the putative SUMO conjugation motif with high probability. SUMOylation probability scores were calculated using the SUMOplot™ analysis program, and those with high probability are shown below the amino acid sequence. The underline indicates the C-terminal-specific sequence of PFR1, used as an antigen for a specific antibody. (TIF) [file pone.0037183.s003.tif]
